# Epidemiological analysis of septic shock in the plateau region of China

**DOI:** 10.3389/fmed.2022.968133

**Published:** 2022-09-14

**Authors:** Qianwei Li, Wenzhao Chai, Xiaoting Wang, Li Cheng, Xin Cai, Jianlei Fu, Wenjun Pan, Guoying Lin

**Affiliations:** ^1^Department of Critical Care Medicine, Tibet Autonomous Region People's Hospital, Lhasa, China; ^2^Department of Critical Care Medicine, Peking Union Medical College Hospital, Peking Union Medical College & Chinese Academy of Medical Sciences, Beijing, China

**Keywords:** high altitude, septic shock, incidence, mortality, risk factors

## Abstract

**Purpose:**

Little epidemiological data exist on patients with severe infection in the plateau region of China, and the data that do exist are lacking in quality. Using the medical records of patients with severe infection in the Department of Intensive Medicine (intensive care unit; ICU) of the People's Hospital of Tibet Autonomous Region, this study analyzed the epidemiological and clinical characteristics of patients with septic shock in plateau area (Tibet), with the ultimate aim of reducing the incidence and mortality from this condition.

**Methods:**

Clinical data on 137 patients with septic shock in the studied ICU from November 2017 to October 2019 were retrospectively analyzed using SPSS, Version 21.0.

**Results:**

Among the 137 patients with septic shock, there were 47 survivors and 90 in-hospital or post-discharge deaths. There were 91 male patients and 46 female patients. The incidence of septic shock was 11.3%, and mortality rate was 65.7%. Median age was 55 years old, median APACHE-II score on the day of admission was 17, median SOFA score was 11, and median number of organ injuries was one. APACHE-II score (*P* = 0.02), SOFA score (*P* < 0.001), and the number of organ injuries (*P* < 0.001) were higher among patients who died than among survivors. The infections were mainly pulmonary and abdominal, and the main pathogen was gram-negative bacteria.

**Conclusion:**

The incidence and mortality of septic shock in ICU wards in Tibet are very high. The APACHE-II score, SOFA score, and the number of organ damage on the first day after diagnosis are independent risk factors for septic shock. To some extent, this study reflects the epidemiological characteristics of septic shock in the plateau region of China (≥ 3,650 m above sea level) and provides data that can support the prevention and treatment of sepsis in the future. More and deeper epidemiological studies of septic shock are necessary.

## Introduction

Sepsis is a life-threatening organ dysfunction caused by the host's response to an infection (1). Sepsis is one of the main fatal diseases for patients in intensive care units (ICUs) (1, 2) and is associated with a significant disease burden and negative economic impact (3). The number of sepsis patients worldwide exceeds 19 million each year, six million of whom die. Worldwide, the sepsis mortality rate exceeds 25%, and about three million patients with sepsis who survive have cognitive impairment (4).

At present, the world pays significant attention to septic shock, and research on the epidemiology of septic shock has gradually increased; however, most of these studies have been conducted in developed countries, primarily in Europe and the United States, and only a few studies on the incidence of sepsis in ICUs have been carried out in mainland China. Research on the sepsis mortality in the whole world has concentrated on tertiary teaching hospitals in large cities (5), and data are particularly scarce for plateau areas (≥ 3,650 m above sea level), where the economy is less developed and the distribution of medical resources is unbalanced. Tibet is located in China's southwestern frontier, which has a large area and is sparsely populated. Overall, transportation in the region is inconvenient, and the distribution of medical resources is unbalanced. Most primary hospitals in this area do not have the ability to identify and treat patients with septic shock, who need to be treated in a Grade A hospital. The Department of Critical Care Medicine of the People's Hospital of Tibet Autonomous Region is the first established relatively complete department of its kind in Tibet; thus, most patients with severe infections are treated here. This study first analyzed the medical records of patients with septic shock admitted to this ICU in Tibet which is one of the typical plateau area around world to clarify their epidemiological and clinical features, with the ultimate aim of preventing septic shock and reducing sepsis. The current incidence of shock and the present case fatality rate can serve as reference values for these efforts.

## Materials and methods

### Sample and data

We used data on 137 patients with septic shock admitted to the Department of Intensive Medicine of the People's Hospital of Tibet Autonomous Region from November 2017 to October 2019.

Patients diagnosed with septic shock in the ICU or during hospitalization were included in the study. As for diagnostic criteria, all included patients met the criteria for the diagnosis of septic shock specified in Sepsis 3.0, which was issued jointly by the Society of Critical Care Medicine and the European Society of Intensive Care Medicine in 2016 (6), patients with infection or suspected infection who develop sepsis-related sequential organ failure, as determined by the Sequential Organ Failure Assessment (SOFA) score increasing ≥ 2 points from baseline, can be diagnosed as having sepsis. Septic shock is persistent hypotension caused by sepsis. Patients with septic shock require vasoactive drugs to maintain a mean arterial pressure ≥ 65 mmHg (1 mmHg = 0.133 kPa) and blood lactate concentration > 2 mmol/L after full-volume resuscitation. Patients under 15 years of age and those with <24 h of hospitalization were excluded from the study.

### Research methods

#### Data collection

Data were collected on associated infection indicators [WBC, PLT, procalcitonin (PCT), and CRP], as well as patient age, sex, outcome of hospitalization, major diagnosis, site of infection, underlying disease, pathogens, number of organ injuries, Acute Physiology and Chronic Health Enquiry (APACHE-II) score on the first day of admission, Sequential Organ Failure Assessment (SOFA) score, use of continuous renal replacement therapy (CRRT), and total mechanical ventilation time. At the hospital level, we collected data on the total number of patients admitted and the total number of patients with septic shock.

#### Zero-time point

The zero-time point was set as the time when the patient was admitted to the ICU for septic shock or the time of diagnosis of septic shock in the ICU. For patients with multiple infections, only the time of the first septic shock was included in the analysis.

#### Data analysis

The data were analyzed using SPSS, Version 21.0 software. Data on normally distributed variables are expressed as means and standard deviations. For non-normally distributed variables, we present medians and interquartile ranges (IQRs). The independent *t*-test was used for normally distributed variables, and the Mann–Whitney *U* test was used for non-normally distributed variables. For categorical variables, we adopted the chi-square test or Fisher's exact test. We conducted a multiple logistic regression analysis to determine the independent predictors of in-hospital mortality in patients with septic shock, and results are presented as odds ratios and the corresponding 95% confidence intervals. Variables with *P* ≤ 0.2 in the univariate analysis, such as demographic characteristics, underlying diseases, disease severity, admission status, and prognosis, were included in the multivariate model. The Hosmer–Lemeshow test was used to evaluate the goodness of fit of the regression model. Unmatched comparisons were performed, and all significance tests were two-tailed, with *P* <0.05 considered statistically significant.

#### Related explanations

1. Patients with septic shock were divided into those who died and those who survived depending on whether they died within 28 days of the zero-time point.
2. Because of regional and religious customs, the families of critically ill patients in the study area relatively frequently request the patients' discharge. Telephone follow-up indicated that more than 98% of self-discharged patients died within 28 days of discharge; therefore, in this study, self-discharged patients were considered to have the outcome of death within 28 days. These cases were combined with in-hospital deaths for total mortality.

## Results

### Population characteristics and incidence of septic shock

Table 1 shows that, from November 2017 to October 2019, 1,216 patients were admitted to the studied ICU, and admissions for septic shock accounted for 137 patients. A total of 47 patients with septic shock were in the survivor group. The incidence of septic shock was 11.3%, and the death of 90 patients corresponded to a case fatality rate of 65.7%. Compared with related reports in China and elsewhere, this case fatality rate is notably high (6). Among the 137 patients with septic shock, there were 91 male patients and 46 female patients, and the median age was 55 years old (QR: 45.5–68.5). There were 56 patients aged over 60 years, and 39 of these patients died, accounting for 42.2% of all deaths. The *P* value for the comparison of the survivors and deaths was > 0.05, indicating no statistically significant difference (see Table 1). About half (47.4%) of the patients with septic shock had chronic diseases, mainly cardiovascular and respiratory diseases. On the day of admission, median APACHE-II score was 17 points (IQR: 12–22), median SOFA score was 17 points (IQR: 8–13), median number of organ injuries was one (IQR: 0–3), and median duration of mechanical ventilation was 55 h (IQR: 11.5–154). There were significant differences between those who survived and those who died in median APACHE-II score (*P* = 0.02), median SOFA score (*P* < 0.001), and median number of organ injuries (*P* < 0.001), but not in median duration of mechanical ventilation (*P* = 0.889). See Table 2 for further baseline information.

**Table 1:** Percentage distribution of age by outcome of septic shock.

**Age**	**Total** **(*n* = 137)**	**Survival group** **(*n* = 47)**	**Death group** **(*n* = 90)**	***P* value**
Oct-40	25 (18.2%)	10 (21.3%)	15 (16.7%)	0.672
41–60	56 (40%)	20 (42.6%)	36 (40%)	
>60	56 (40.9%)	17 (36.2%)	39 (42.2%)	

**Table 2:** Population characteristics by septic shock outcom.

**Variable**	**Total**	**Survivors**	**Deaths**	***P* value**
	**(*n* = 137)**	**(*n* = 47)**	**(*n* = 90)**	
Age, median (IQR)	55 (45.5–68.5)	54 (45–67)	55 (45.5–69.5)	0.812
**Gender**				
Male	91 (66.4)	30 (21.9%)	61 (44.5%)	0.705
Female	46 (33.6)	17 (12.4%)	29 (21.2%)	0.589
APACHE-II score (IQR)	17 (12–22)	13 (9–20)	17 (13.75–23.25)	0.02
SOFA (IQR)	11 (8–13)	9 (7–11)	11 (9–14)	0
Number of organ injuries (IQR)	1 (0–3)	0 (0–2)	2 (1–3)	0
**Mechanical ventilation**				
Yes	117 (85.4%)	37 (27%)	80 (58.4%)	0.13
No	20 (14.6%)	10 (7.3%)	10 (7.3%0)	0.427
Time of use of mechanical ventilation	55 (11.5–154)	63 (4–218)	51 (15.25–144)	0.889
**CRRT**				0.164
Yes	128 (93.4%)	82 (59.9%)	46 (33.6%)	0.164
No	9 (6.6%)	1 (0.7%)	8 (5.8%)	0.037
WBC	11 (5–17.4)	11 (7–15.9)	11.1 (6.23–18)	0.73
PLT	106 (53–177)	124 (66–210)	97.5 (38.5–226.9)	0.117
PCT	3(0.31–21.54)	0.82 (0.16–16.5)	5.08 (0.59–32.46)	0.042
CRP	136.24 (66.3–220.7)	134.61 (51.97–192.66)	136.24 (68.24–253.13)	0.103
**Basic diseases**				
Cardiovascular diseases	23 (16.8%)	9 (6.6%)	14 (10.2%)	0.234
Respiratory diseases	16 (11.7%)	3 (2.2%)	13 (9.5%)	0.593
Endocrine diseases	7 (5.1%)	4 (2.9%)	3 (2.2%)	0.142
Cardiovascular + endocrine	8 (5.8%)	2 (1.5%)	6 (4.4%)	0.468
Diseases				
Biliary system	4 (2.9%)	0	4 (2.9%)	0.568
Gastric and duodenal diseases	2 (1.5%)	0	2 (1.5%)	0.191
Disease				
Chronic diseases	1 (0.7%)	0	1 (0.7%)	0.303
Tumor and immune diseases	4 (2.9%)	1 (0.7%)	3 (2.2%)	0.163
Basic diseases	72 (52.6%)	28 (20.4%)	44 (32.1%)	0.399

### Infection site and pathogen

The lungs (40.9%) and abdominal cavity (15.3%) were the most common sites of infection in patients with septic shock, as shown in Table 3. Among 34 patients, the infection site comprised two or more mixed infections. There were six cases of influenza infection (4.4%; mortality = 100%). Among the 137 patients with microbial culture, 38 (27.7%) were isolated from gram-negative bacteria, 13 (9.5%) were isolated from gram-positive bacteria, and 10 (7.3%) were isolated from fungi. In 46 cases (33.6%), there was a mixed infection of multiple bacteria (see Tables 3, 4).

**Table 3:** Distribution of type septic shock infection by outcome.

	**Total**	**Survival group**	**Death group**	***P* value**
	**(*n* = 137)**	**(*n* = 40)**	**(*n* = 97)**	
Pulmonary infection	56 (40.9%)	19 (40.4%)	37 (41.1%)	0.267
Intraperitoneal infection	21 (15.3%)	9 (19.2%)	12 (13.3%)	0.18
Urinary infections	1 (0.7%)	1 (2.1%)	0	0.083
Blood flow infection	6 (4.4%)	0	6 (6.7%)	1
Intracranial infection	3 (2.2%)	2 (4.3%)	1 (1.1%)	0.999
Mixed infection	34 (24.8%)	7 (14.9%)	27 (30%)	0.115
Other	16 (11.7%)	9 (19.1%)	7 (7.8%)	0.015

**Table 4:** Distribution of pathogens in septic shock by outcome.

	**Total**	**Survival group**	**Death group**	***P* value**
	**(*n* = 137)**	**(*n* = 40)**	**(*n* = 97)**	
Negative	30 (21.9%)	10 (7.3%)	20 (14.6%)	0.296
G−	38 (27.7%)	11 (8%)	27 (19.7%)	0.899
G+	13 (9.5%)	8 (5.8%)	5 (3.6%)	0.413
Fungi	10 (7.3%)	3 (2.2%)	7 (5.1%)	0.30
Mixed infection	46 (33.6%)	15 (10.9%)	31 (22.6%)	0.766

### Mortality for septic shock

Among the 137 patients with septic shock, 90 died in the hospital or after ceasing treatment because of excessive illness or folk customs. The overall mortality was 65.7%.

Median APACHE-II score in the first 24 h after diagnosis of septic shock was higher among those who died (17; IQR: 13.75–23.25) than among those who survived (13; IQR: 9–20; *P* = 0.02). Likewise, for SOFA score, the median was higher among patients who died (11; IQR: 9–14) than among those who survived (9; IQR: 7–11; *P* < 0.001). The median number of organ injuries was also higher among those who died (2; IQR: 1–3) than among those who survived (0; IQR: 0–2; *P* < 0.01). In terms of the infection index, median PCT was significantly higher among those who died (5.08; IQR: 0.59–32.46) than among those who survived (0.82; IQR: 0.16–16.5; *P* < 0.05; see Table 2).

### Risk factors for death from septic shock

The death of patients with septic shock was used as the dependent variable, and indicators that were meaningful in the univariate analysis were used as the independent variables. The multivariate logistic regression analysis showed that APACHE-II score, SOFA score, and the number of organ injuries were statistically significantly associated with the risk of death from septic shock. Table 5 presents the analysis of the independent risk factors for death from septic shock.

**Table 5:** Risk factors for septic shock death.

**Risk factors**	***M* (IQR)**	***P* value**	**OR (95% CI)**
APACHE-II score	17 (12–22)	0.002	1.099 (1.036–1.167)
SOFA score	11 (8–13)	0	1.198 (1.073–1.338)
Number of organ injuries	1 (0–3)	0	1.929 (1.39–2.678)

## Discussion

The incidence of septic shock in the ICU was 11.3% (137 cases of septic shock/1,216 total ICU admissions) in this study. The lungs and abdomen were the most common sites of infection; the most frequently observed pathogen was gram-negative bacteria, and most of the patients had mixed infections. There was no significant difference in mortality between patients with underlying diseases and those without underlying diseases (mortality: 32.1% vs. 33.6%, *P* > 0.05); the case fatality rate for septic shock was high, at 65.7% (*n* = 90). APACHE-II score, SOFA score, and the number of organ injuries were higher among patients who died than among those who survived, and the multivariate analysis showed that these variables were independent risk factors for death from septic shock.

Our results are similar to Bin Du's descriptive analysis of sepsis-related mortality in China (7). Sepsis-related mortality was significantly lower in areas with higher disposable income. Increasing mean years of education was negatively associated with sepsis-related mortality. The incidence and mortality found in this study were higher than the those reported by two multi-center surveys of ICUs in mainland China, which reported the total case fatality rate of patients with severe sepsis to be 48.7% and 33.5%—significantly higher than the mortality for severe sepsis in developed countries (8). In Australia and New Zealand, the hospital case fatality rate for severe sepsis declined from 35% in 2000 to 18.4% in 2012; in the United States, the mortality for sepsis (defined by ICD-9-CM) dropped from 27.8% in 1979 to 18.4% in 2012 and then 17.9% in 2000 (9).

There are several reasons that may explain why the incidence and mortality of sepsis is high in the plateau area of China. First, sepsis is a host reaction disorder caused by infection, leading to life threatening organ dysfunction (10). Acute circulatory dysfunction caused by insufficient oxygen delivery and / or impaired cellular oxygen utilization is a major cause of death due to septic shock (11). Microcirculation plays an important role in tissue perfusion; it can ensure that oxygen is delivered to tissues, exchange nutrients and wastes; and regulate inflammation and coagulation. Plateau area is a special geographical environment, with the increase of altitude and the decrease of air pressure, the oxygen content in the air gradually decreases, resulting in a series of hypoxic reactions. The low oxygen content in the atmosphere can significantly reduce the oxygen content of human arterial blood (12). Whether living at high altitude for a long time or entering the plateau rapidly, chronic hypoxia or acute hypoxia, and septic shock, microcirculation is always characteristically changing. The permeability of endothelial barrier plays an important role in maintaining humoral homeostasis and regulating the physiological functions of tissues and organs. Endothelial cells are the main component of microvascular permeability barrier, and endothelial glycocalyx is a layer of glycosaminoglycan and related proteoglycan lining the vascular lumen. In the case of infection and hypoxia (13), the glycocalyx of endothelium degrades greatly, and the permeability of endothelium increases, which leads to the leakage of fluid into the tissue space, resulting in tissue edema; endothelial cells can also stimulate leukocytes to release a large number of inflammatory mediators (TNF-α, IL etc.), at the same time, endothelial cells are over apoptotic, stimulate the expression of adhesion molecules, and release oxygen free radicals, further amplify the role of leukocytes. The phospholipid membrane of apoptotic endothelial cells is exposed to the blood, forming a coagulation promoting reaction surface and activating the coagulation system (14). Hypoxia in high altitude environment will further stimulate capillary cell damage, aggravate microcirculation and cell dysfunction, and eventually lead to organ dysfunction and increased risk of death (12). Second, the Intensive Care Department of the People's Hospital of Tibet Autonomous Region was established relatively recently—in 2008. This hospital is more advanced than other hospitals in the region and treats most patients with severe infections. Therefore, the number of patients with septic shock is higher at the People's Hospital of Tibet Autonomous Region than that at other hospitals in the region, which may contribute to explaining the high incidence observed in the present study (15). Third, many patients can still be treated, despite being critically ill; however, these people are sometimes self-discharged because of religious beliefs and customs. Additionally, some families of critically ill patients choose to abandon treatment because of the high cost of hospitalization. When they were followed up, the great majority of patients who were self-discharged for these reasons had died. This is also a reason for the relatively high mortality observed in this study. This finding should stimulate autonomous regions, counties, townships, and towns to increase public awareness of disease, strengthen the training of local doctors, and further improve the population's awareness of disease prevention and control. Fourth, in the present study, those aged over 60 years accounted for about 40.9% of the cases and 42.2% of all deaths. The overall population is aging, and older adults are more heavily affected by sepsis, compared with their younger counterparts (4). In older age, organ function declines, immunity is reduced, and the number of underlying diseases increases. After infection, septic shock also tends to be more severe in older adults than in younger people, and it is more difficult to reverse the effects in those of older ages. This may be an additional reason for the high incidence seen in this study. Fifth, we found that 41–60-year-olds made up about 40% of all people with septic shock, and this age group also accounted for 40% of all deaths. The development of bad habits in daily life, such as staying up late, excessive drinking, smoking, the lack of exercise, and other harmful behaviors, worsen the cardiovascular health of adults in middle age. The incidence rates of cardiovascular diseases, endocrine diseases, respiratory diseases, tumors, and other diseases reveal increasing trends, whereas immunity is declining, which increases the susceptibility to infection (16). Higher risk of infection, in turn, increases morbidity and mortality among middle-aged people.

Our study is consistent with the results of some international multi-center studies. We found that the infection site of septic shock is mainly the lungs and abdominal cavity, followed by bloodstream infection. This finding suggests that, when the cause of septic shock is unclear, lung and abdominal infections should be given priority, but other infection sites should not be neglected. Previous studies have reported that 70–80% of cases of septic shock caused by pulmonary infection are acquired in the community, which requires emergency and primary care to become key targets for awareness raising and early management (17). Unbalanced distributions of medical sources and insufficient ratios of medical staff to patients in hospitals are associated with higher incidence of hospital-acquired pneumonia (18). Common causes of nosocomial infection are low levels of awareness of hospital infection prevention and control and poor hand and environmental hygiene. Additionally, critically ill patients in the ICU often require sedation, analgesia, and open airways, all of which reduce their immune defenses. At the same time, because of improper care, bed rest may cause lung consolidation, atelectasis, and gastric juice reflux. If measures are not in place for the prevention and control of hospital infection and bacteria are spread, a sudden increase in bacterial load may result in fatalities (19, 20). Shock induced by abdominal infection is more common in cases of delayed removal of the infected foci and inadequate surgical drainage, resulting in excessive bacterial load and increased mortality. Therefore, early diagnosis of sepsis, timely intervention, drainage of postoperative infections, and reduction of bacterial load are extremely important and should be the focus of continued efforts in the future. Bloodstream infection has a very high mortality among ICU patients—as high as 100% in this study. Therefore, close attention should be paid to aseptic operations during surgeries. In addition, daily skin disinfection should be standardized to reduce the possibility of retrograde bacterial infection.

The results of this study suggest that the pathogen was mainly gram-negative bacteria, which is similar to the results of other research in China. To reduce bias, this study examined pathogenic specimens within 24 h of the diagnosis of septic shock, which facilitates the accurate identification of the pathogenic microorganisms of septic shock and can further guide the correct use of antibiotics. It is not uncommon for the abuse of antibiotics to cause microbial resistance; therefore, the sensible selection of antibiotics is another area requiring continuous efforts in the future.

This study showed that APACHE-II score, SOFA score, and the number of organ injuries were independent risk factors for death from septic shock. Among infection indicators, PCT showed a strong positive correlation. APACHE-II score, which was developed as early as the 1980s, has been widely used in clinical practice. This score performed well in predicting the risk of hospital death, and previous studies have also reported that a higher APACHE-II score often indicates a higher risk of hospital death (21). Increases in SOFA score and in the number of organ injuries often indicate more serious organ failure (22), which significantly affects the patient's ultimate outcome. Therefore, in clinical practice, patients with higher scores and higher infection indexes should receive strict dynamic observation and monitoring to slow the progress of the condition and formulate a reasonable and refined treatment plan.

## Conclusion

The incidence and mortality of septic shock in ICU wards in Tibet are very high. The APACHE-II score, SOFA score, and the number of organ damage on the first day after diagnosis are independent risk factors for septic shock. To a certain extent, this study reflects the epidemiological characteristics of septic shock in plateau areas (3,650 m and above), and provides certain data support for the prevention and treatment of sepsis in this region in the future. In the future, it is necessary to carry out more, more extensive and more in-depth epidemiological studies of septic shock.

## Data availability statement

The raw data supporting the conclusions of this article will be made available by the authors, without undue reservation.

## Ethics statement

Written informed consent was obtained from the individual(s) for the publication of any potentially identifiable images or data included in this article.

## Author contributions

QL is mainly responsible for writing and statistics of articles. LC, XC, and JF are mainly responsible for data collection. GL, WP, WC, and XW are responsible for guiding the collaboration. All authors contributed to the article and approved the submitted version.

## Funding

This work was funded by Natural Science Foundation of Tibet Autonomous Region (XZ2020ZR-ZY15Z) and Science and Technology Plan Project of Tibet Autonomous Region (XZ202101YD0019C).

## Conflict of interest

The authors declare that the research was conducted in the absence of any commercial or financial relationships that could be construed as a potential conflict of interest.

## Publisher's note

All claims expressed in this article are solely those of the authors and do not necessarily represent those of their affiliated organizations, or those of the publisher, the editors and the reviewers. Any product that may be evaluated in this article, or claim that may be made by its manufacturer, is not guaranteed or endorsed by the publisher.
